# The impact of parent involvement on improving participation of children born preterm: The story in the baseline

**DOI:** 10.1016/j.conctc.2022.100942

**Published:** 2022-06-14

**Authors:** Hazel Killeen, Dana R. Anaby

**Affiliations:** a College of Medicine, Nursing and Health Sciences, Áras Moyola, National University of Ireland, Galway, Galway, H91 TK33, Ireland; b McGill University, School of Physical and Occupation Therapy, 3630 Promenade Sir-William-Osler, Hosmer House Rm. 302, Montreal, Quebec, H3G 1Y5, Canada

**Keywords:** FAD, McMaster Family Assessment Device, PEM-CY, Participation and Environment Measure for Children and Youth, PREP, Pathways and Resources for Engagement and Participation, COPM, Canadian Occupational Performance Measure

## Abstract

**Background:**

Preterm birth continues to be a major public health challenge that has long term consequences on participation into adulthood. However, little is known about effective interventions to improve the participation of children born preterm.

**Methods:**

This study gathered initial evidence on the usefulness of a goal-focused, environmental-based approach (Pathways and Resources for Engagement and Participation (PREP)) in improving the participation of children born preterm, and living in Ireland. Three school-age boys (6–7 years old) with a history of preterm birth participated in the 12-week PREP intervention. A 36-week single-subject AB design was employed and replicated across 3 different participation goals within each child and across 3 children. Activity performance was measured repeatedly, through parental involvement, using the Canadian Occupational Performance Measure (COPM), providing 9 individual outcome trajectories. Visual inspection and mixed-effects segmented regression were used.

**Results:**

Goals were selected from various participation domains and settings. Throughout the baseline phase, once goals were set, significant improvements in activity performance were observed for all participants (t = 14.06, p < 0.001). Further clinically significant improvements (2.58 on the COPM) for all 9 participation goals were seen in overall performance during the intervention phase. These changes remained at follow-up.

**Conclusions:**

Findings support family-centered practice and draw attention to the power of goal setting in improving participation within this context. Challenges with single-subject design with this population were also highlighted. Results demonstrate the potential impact of parent involvement when using an environmental-based approach to improve the participation of this underserved population.

## Highlights

• Improvement in participation in children born preterm, without physical disabilities.
• Challenges of single-subject design with this population.
• Environmental-based strategies show effectiveness in addressing participation goals.
• Environmental-based strategies may promote capacity building in parents.
• Studies focusing on the impact of goal setting and parental involvement are needed.

## 1 Introduction

Evidence on the participation patterns of young children born preterm is emerging, yet scarce. These children have significantly lower adaptive behavior in comparison to age-matched term-born infants [1] which in turn has a negative effect on their level of participation, particularly in play [2]. Parents of children born very preterm also attend community activities less regularly and report more barriers in participation than term-born controls [3]. Long-term repercussions are characterized by limited participation into adolescence, in comparison to full-term youth [4], especially in physical and skill-based activities [5].

Goal-directed family-centered interventions that are activity-based and occur in the child's natural environment are considered best practice for improving participation [6]. Numerous studies on goal-setting have, however, found limitations with goal quality and processes, not least in relation to incorporating the client's perspective [7,8]. According to Self-determination theory, families' motivation to engage in the goal setting process is influenced by their basic psychological need for Autonomy, Relatedness and Competence [[9], [10], [11]]. Parents' who become actively involved in goal setting have been shown to have increased feelings of competence and empowerment and demonstrate improved partnerships with professionals [12,13] likely increasing their self-determination to achieve their family goals [14,15]. In a qualitative study of a newly developed collaborative goal setting tool, parents described their experience of using the tool as empowering, reporting increased ownership or independence in the family goal setting process [16]. Adult collaboration with children during goal setting has been found to strengthen the child's self-esteem and bring attention to the child's dreams and wishes. Child-oriented goal setting has also been perceived as a contributing factor to child participation [17]. Few, if any, studies have explored the impact of collaborative goal-setting on the participation of families of children born preterm.

As the environment has been consistently shown to influence the participation of children with disabilities [[18], [19], [20]], a range of innovative environmental approaches have emerged. Examples include context-therapy for young children with cerebral palsy [21]; TEAM approach (Teens making Environment and Activity Modifications) for youth with developmental disabilities [22], as well as the Pathways and Resources for Engagement and Participation (PREP) practice model designed for individuals across different ages and abilities [23], found effective among adolescents with physical disabilities, living in Canada [24]. Despite concerns, however, that environmental conditions may mitigate or aggravate the impact of biological risk factors on child development [25], the association between preterm infants and their social and familial environment is understudied.

This study aims to explore the initial usefulness, of an environmental-based approach (PREP), that involves parental involvement through ongoing setting and monitoring of family centered goals, in improving the participation of school-aged children born preterm. The PREP was chosen because it is a generic, inclusive and broad practice model designed for individuals with any type of disability across the life span and is applicable to any activity of choice [23]. The former preterm children in this study were without a persistent physical disability (e.g., cerebral palsy), and living in Ireland.

## 2 MATERIAL and METHODS

### 2.1 Design

Initially, an interrupted time series design with multiple baselines across participation goals was planned. The design was to be completed over a 20-week period (4-week baseline phase, 12-week intervention period and a 4-week follow-up). The intervention was to be implemented for three individualized participation goals and introduced at varying time points. By varying the length of the baseline phase across participation goals, extraneous variables such as maturation and carry-over effects would be controlled for (Backman & Harris, 1999). Multiple baseline designs have also been recognized as one of the most feasible and methodologically rigorous approaches to evaluating intervention effects (Logan et al., 2008). During the baseline phase however, once goals were collaboratively set with families, families began to make focused efforts to address their goals resulting in significant improvements during the baseline phase. It was no longer feasible therefore to have different lengths of baselines and the overall research design needed to be adapted. Possible reasons for the families’ motivation and self-direction during the baseline phase will be discussed later in the paper.

With ethical approval, the research design was adapted to a single-subject AB research design [26] employed over a period of 36 weeks: 4-week baseline, 12-week intervention, and follow-up on week 20 and 36. Following Ganz and Ayres’ recommendations [27], this design was replicated, as planned, across 3 different participation goals within each child and across 3 children, resulting in 9 individual trajectories representing change in participation. Performance in identified participation goals were rated by the parent twice a week throughout the entire phases of the study; baseline, intervention and follow-up. Overall, the baseline assessment ran for 4 weeks (providing 8 outcome ratings), the intervention phase for 12 weeks (24 ratings) and then follow-up (2 ratings), resulting in 34 data-points per trajectory/goal. Collecting multiple ratings allows to detect if change occurs, when it occurs and to what extent. It also allows for testing changes in trends statistically.

### 2.2 PREP intervention

The PREP, a client-centered intervention to improve participation in chosen activities by removing environmental barriers and coaching parents, was delivered individually for each child/family. It involved five steps, Make goals, Map a plan, Make it happen, Measure process and outcomes and Move forward [28], targeting three chosen activities or participation goals over a period of 12 weeks. Four-week intervention was designated for each goal and involved up to 4 h of therapy time for a total of up to 12 h overall (4 h X 3 goals). An initial 2-h session took place with each parent and child in their home, to administer pre-intervention assessments related to participation patterns (PEM-CY; Participation and Environment Measure for Children and Youth), family functioning (FAD; Family Assessment Device) and demographic information. During the session, the Canadian Occupational Performance Measure (COPM) was used to empower parents and child to select 3 participation goals. The semi-structured format of the COPM facilitated the therapist to engage in a family-centered interview with the parent. The interview focused on identifying areas of priority for their child's participation with a focus on what was meaningful and important to both the parents and child. The therapist and parent together translated these priorities in to three child-oriented goals. The parents were then asked to rate each goal on a scale from 1 to 10 in relation to how important they were to the family, what they perceived their child's current level of performance to be, and how satisfied they were with that level of performance. In this study, parents and children were also asked to select child-oriented goals [29] that 1) were important to them both yet considered difficult to carry out, and 2) were not being addressed through any other services. Participation goals were related to any of the COPM domains (self-care, leisure, productivity) across a range of settings: home, school and/or community.

Following the initial COPM interview, including goal-setting (PREP step 1), a 4-week baseline phase commenced. During this phase, parents' rated their child's performance for each goal by phone with the therapist twice a week, using the COPM performance scale. No structured intervention was offered. Following this phase, the PREP intervention was introduced. For each goal/selected activity, the therapist and parents jointly identify environmental barriers and explore solution-based strategies to modify these barriers while building on existing supports. For example, searching for information, advocating for child's inclusion, reaching out to community resources and working jointly with relevant stakeholders in order to facilitate participation. Parents' perception of performance for each participation goal continued to be monitored bi-weekly by phone by the therapist using the COPM throughout the intervention phase. During the follow-up phase, 4 weeks and 20 weeks following the completion of intervention for all goals, performance (COPM) and participation patterns (PEM-CY) were measured.

### 2.3 Procedures

Ethics approval (*C.A 1637*) was granted by the Clinical Research Ethics Committee, Galway University Hospitals, Galway, Ireland in November 2016. The study commenced in May 2017 and was completed in March 2018. Two experienced occupational therapists reviewed the PREP manual and completed a 2-h training on the approach through a webinar and an online meeting. Ongoing expert support was provided throughout the entire study. To further ensure treatment fidelity, all intervention strategies documented by the therapists were reviewed and found to adhere to the principles of the PREP; that is, focusing solely on modifying aspects of the environment (with no remediation of impaired body functions). Each participant was randomly assigned to one of the two therapists. Informed written consent was obtained from all parents.

### 2.4 Measures

At baseline only, family functioning was measured using the 7 subscales of the FAD (e.g., problem solving, communication, general Functioning subscale) [30]. The FAD includes 60 statements rated on a 4-point scale (1 = strongly agree; 4 = strongly disagree). Mean scores range from 1 to 4 where higher scores indicate family functioning is poorer or unhealthy/pathological. The cut-off score for the FAD subscales ranges from 1.9 to 2.3; thus, a score greater than ∼2 suggests a problem in or unhealthy family functioning. The FAD demonstrated adequate test-retest reliability (0.66–0.75) and validity [31] and has been used effectively among school-age clinic-based samples [32].

A demographic questionnaire gathered information on the child and their family. The complexity of child's condition was documented using a list of 13 health problems (e.g., orthopedic/movement, vision impairment) and 11 functional issues (e.g., learning new information, using hands to do activities), checked off by the parents.

Participation goals were established and parents' perceptions of performance were repeatedly measured using the COPM [33]. The COPM is a standardized assessments that uses a semi-structured interview to guide parents and their child to identify activities that are important to them and rate level of performance on a 10-point scale (1 = unable to perform to 10 = performs extremely well). The COPM is a reliable and valid measure that can detect clinically significant changes in performance (i.e. an increase of at least 2 points on the scale), serving as an excellent responsive outcome measure [[34], [35], [36]].

As a secondary outcome, Participation patterns and environmental barriers/supports were measured at baseline, post-intervention (week 20) and during follow-up (week 36) using the PEM-CY [37]. It assessed participation in 25 activities across three settings; home (10 activities), school (5 activities) and community (10 activities). Parents rated their child's participation *frequency* (1 = never to 7 = daily), level of *involvement* (1 = minimally involved to 5 = very involved) and number and type of environmental barriers/supports (e.g., physical, social/attitudinal, availability of programs and equipment) affecting participation. The PEM-CY is a reliable and valid measure, with moderate to very good internal consistency (α = 0.59 to 0.91) and test–retest reliability (ICC = 0.58 to 0.95) [37].

### 2.5 Participants

Participants were sampled from a previous Irish study (n = 44) that explored the participation of young children born preterm [1]. As with the original study, inclusion criteria included children born preterm and very low birth weight (less than 1500 g and/or less than 30-week gestation) as defined by the Vermont Oxford Network [38]. These children were purposively chosen from the previous study as it was difficult to identify children who met the above criteria and did not have a diagnosis of physical or intellectual disability [1].

In the original study, participants’ adaptive functioning was assessed using the Adaptive Behavior Assessment System, Second edition (ABAS-II) [39] which provides standardized scores (mean = 100, SD = 15). Specific to this study, participants were required to be one standard deviation or lower from the norm on the ABAS-II to ensure participants included presented with functional difficulties that would require intervention. In the original study, this cohort of children between 2 years and 5 years 6 months of age did not, however, demonstrate significant differences in their diversity and intensity of childhood participation relative to their full-term peers. As the literature shows significant deficits in the participation of adolescents who were born preterm [4], it is thought that in the early years participation may be scaffolded by the parents or other adults in the home and preschool setting. Challenges may become more evident at school-age once tasks become more complex and there is less parent-involvement. In the country where this study was conducted little to no intervention was received by this cohort of preterm infants at school age. None of the children in the current study were receiving rehabilitation services at the time of the study.

### 2.6 Sample characteristics

Three male school-aged children, 6–7 years old, living in a dual parental household in Ireland participated. All children attended regular primary schools with learning support and one child had access to a special needs assistant. Parents' ages ranged from 37 to 47 years old, and their level of education varied from high school diplomas to bachelor's degrees. Children's levels of functional behavior based on the ABAS-II (General Adaptive Composite) ranged from a standard score of 53–85. Based on the FAD, all three families demonstrated relatively healthy/good family function in most of the subscales. Table 1 describes each child's health condition and family functioning.

**Table 1:** Sample characteristics

Participant	# Health Conditions	Health conditions	# Functional Issues	Functional Issues	Family functioning†
1	5	- hearing - speech/language - vision - orthopedic/movement - other	9	- paying attention/concentrating - remembering information - learning new information - communicating with others - reacting to sensation - using hands to do activities - managing emotions - controlling behavior/activity level - seeing	All scores were below the cut-off points indicating healthy/good family function (<1.5), with the exception of family roles (2.36).
2	3	- speech/language - vision - orthopedic/movement	4	- paying attention/concentration - moving around - using hands to do activities - managing emotions - controlling behavior/activity level - seeing	5/7 scores were just below cut-off point (2) and 2/7 scores met the cut-off point, indicating a potential risk for unhealthy/poor family functions.
3	1	- health impairment	5	- paying attention - remembering information - learning new information/activities - using hands to do activities - seeing	All scores were below the cut-off points (<1.7) indicating healthy/good family function, with family roles being close to the cut-off point (2.21).

†Measured using the FAD= Family Assessment Device.

### 2.7 Data analysis

To examine improvement in participation, all plotted trajectories representing change in performance were visually analyzed. To complement visual inspection, which is subjective and susceptible to type I error [26], mixed effects segmented regression was used. This allowed us to model the trajectories of change for three goals within each of the three participants for a total of nine trajectories. Our ability to model full random effects was hampered by the sample size, but we were able to model the random variation of the intervention intercept for goals nested within participants, and the random variation in change over time during treatment among participants. To evaluate whether the intervention conferred clinically meaningful change, we multiplied the estimated bi-weekly rate of change during the intervention period by the average duration of the intervention in the study to provide a point estimate of the expected amount of change incurred during the intervention. Analyses were performed using nlme package [40] in the R statistical programming language [41].

To examine differences in overall participation patterns pre- and post-intervention as well as during follow-up, PEM-CY scores (frequency, involvement, number of environmental supports) were compared descriptively for direction and amount of change.

## 3 Results

Generally, families selected a range of age and developmentally appropriate activities, with children's goals including all areas of participation offered by the COPM; self-care, productivity and leisure activities. The goals related to a variety of environments, including home, school and the community, and were of importance to both parents and children.

**Fig. 1:**
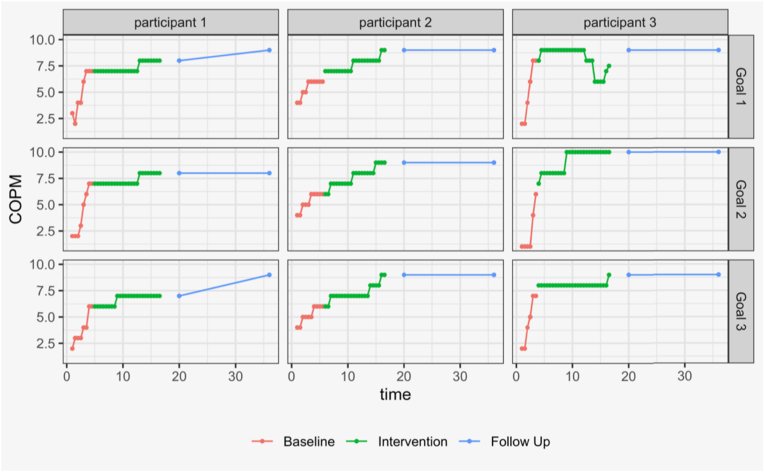
Changes in COPM† performance scores during baseline, intervention and follow-up phases †Canadian Occupational Performance Measure.

Visual analysis indicated that, upon identification of goals during the baseline phase, all three families started to work on achieving their respective goals. Consequently, a large positive change was observed in the baseline phase for all three participants, followed by more modest change in 8/9 goals during the intervention phase, as seen in Fig. 1. Table 2 confirms these findings, the average change during baseline (baseline slope) was 1.7 points per week (p < 0.001). For example, the mother of Child 3 reported that the goal setting process had helped her realize she was assisting her child too much and quickly adjusted her behavior. This resulted in Child 3's performance in participation in self-care on the COPM increase from a score of 2 to a score of 9 by the end of the baseline. Similarly, Child 1's first goal was identified ‘to cycle a bicycle’. He was unable to cycle and had been attempting to ride a bicycle of the incorrect size. In Week 3 of the baseline phase, he received a new bike from his parents that was fitted specifically for him, and he began making progress on this goal.

**Table 2:** Results of the mixed effects model

Parameter	Value	Std. Error	DF	t-value	p-value	Random Effect
Baseline Slope	1.049	0.075	276	14.06	0.000	
Baseline Intercept	1.679	0.232	276	7.23	0.000	
Intervention Slope	0.112	0.057	276	1.96	0.051	0.095
Intervention Intercept	6.475	0.969	276	6.68	0.000	1.63 across clients 0.41 across goals
Residal Error						0.81
DF = Degrees of freedom						

Further clinically significant improvements, as measured by an increase of 2 or more points on the COPM, were seen in all participation goals during the intervention phase. The average biweekly rate of change was 0.112 points and the average length of intervention was 11.5 weeks, which gives an average change of 0.112 × 2 x 11.5 = 2.576 points across participants. This change approached statistical significance (p = 0.051), as shown in Table 2.

During the intervention, the therapist partnered with the families to develop a variety of goal-specific environmental strategies tailored to their unique needs. Table 3 presents examples of strategies used, categorized by environmental domains.

**Table 3:** Examples of Occupational Therapy Environmental Strategies

Goal	Activity modifications	Attitudinal	Institutional	Physical	Social	Temporal/scheduling
To be independent in cycling bike safely with adult supervision		Discussion with physio, to encourage child to maintain interest and to engage in cycling	OT contacted Community OT Services to waitlist child for future cycle camps	Apply stabilizers to wheels and reduce seat height to allow feet to touch the floor; Use of the path within estate initially to follow clear boundary and pathway; brought bike up slight incline, so child starts off on a decline	Sister to cycle ahead of child to highlight obstacles; Parents to discuss group cycle for child and friends in cul de sac area, one parent to supervise group	Cycle on promenade early morning at weekend or at a quieter time in afternoon
To play a school yard game with a minimum of one other child				Allocated section for child to play in yard; More defined markings on yard surface with additional visual markers and greater variety of distances and target markers	Yard duty supervisor to observe and provide help when required only; Small group play in PE time to allow opportunity to model and engage with peers and develop new relationships	
To increase participation in family swimming outing				Checklist of items for bag. Adhere checklist as a key ring to outside of the bag; Easy tie clothing, waistbands, large zipped bag; Use of mirror for dressing and checking clothing post swimming	Family to incorporate swimming into weekly routine; Parents signing child up for local swimming lessons; Ask for assistance if require help for locating items, exit, etc.	Verbal prompting by adult re. use of the clock/time management
To increase independence in homework/writing tasks at home	Snack prior to homework to aid concentration; Child to organize bags/books with parents to encourage independence	Use of motivational tools e.g. first homework, then play time		Dedicate clearly defined space in home for homework, reduce stimuli; Checklist for books to bring from school; Bookshelf over desk for organization of items; Colour code copy to match workbook	1-1 support available for support with organization of material/providing encouragement and guidelines; later progressing to child using homework journal and checklist independently	Agreed time assigned for homework completion, with use of clock/timer to encourage efficiency
To increase independence in dressing tasks as part of swimming activity	Encourage child to organize items in bag and on bench in a sequence		Child waitlisted for swimming group linked to Health Service	Key ring coin for locker attached to zip; Use of changing cubicle to minimize distractions from peers/public; Encourage child to use checklist for dressing/swimming items and reference clock in changing room to encourage time efficiency	Teacher to also encourage independence with dressing tasks before and after school swimming classes; Child to attend local pool weekly with parents	
To increase participation in self-care routines	Breakdown each activity; Introduce upbeat music/movement breaks to increase alertness and readiness for task e.g. use of trampoline, “lucky dip” for chores with child minder	Increase child's motivation for task e.g. gets peers involved, plan picnics, cinema nights.		Use visual schedules and checklists	Mum models and sets up task for child to do e.g. leaves clothes out for the next day, leaves lunch box on counter to put in school bag; Family challenge - Team based chores where team that wins get prize.	
To participate in snack preparation for family once a week	Child makes something he can do himself e.g. peanut butter on rice cake, wrap, baked egg; simple recipes broken down with visuals			Visuals checklists and schedule for cooking	Make Friday night with the child minder after school “kitchen night”; Cue with recipe sheet to keep on task; Siblings worked together well	

On visual inspection of the graphs (Fig. 1), improvements in performance during the intervention phase were maintained at both the 20-week and 36-week follow-up. Further improvement was documented for one child in one activity at week 20 and for another child in two other activities at week 36.

Examining changes in participation patterns using the PEM-CY (secondary outcome), revealed a positive change, especially post-intervention. An improvement in mean frequency of participation in the school and community settings for all participants (0.7–1.8 on a 7-point scale) was seen in both the post-intervention phase (week 20) and at follow-up (week 36). An increase was also observed in mean involvement following the intervention across all settings, ranging from 0.2 to 2.4 (on a 5-point scale). Changes in number of environmental supports were inconsistent.

## 4 Discussion

This study is one of the first to examine an environmental intervention on the participation of young children born preterm, without a persistent physical disability (e.g., cerebral palsy). As was evident in the significant changes observed during the baseline phase across all goals and all participants, once goals were set, all three parents began to work on addressing their child's participation goals. This is worth reflecting on (at both a clinical and methodological level).

Changes may be explained by a number of factors specific to this population and age range of children. For example, the children's young age would have allowed for more intense parent involvement [42]. As many goals were set in controllable environments that were very accessible to parents such as the family home and immediate community, and children had shorter school days than older children, parents may have felt empowered to address the goals and provide regular practice. Our findings suggest that the goal setting process, combined with twice weekly monitoring of goals, in itself may have been beneficial and resulted in positive outcomes during the baseline phase, in isolation of any targeted intervention. This was reported in previous research particularly when participation goals were being targeted [43]. Similarly, a US study of teenagers with developmental disabilities that compared an environmental intervention group to a goal-setting comparison group found that both groups demonstrated significant improvements in self-identified participation goals, problem-solving, knowledge and self-determination [22]. Once intervention was completed in this study, qualitative interviews with parents may have been valuable in revealing the families' deeper motivations.

Findings also highlight the value of parent involvement and their role as experts in their children's intervention, aligned with family-centered principles [44]. Family support and involvement were evident throughout the process and were consistent with the FAD results, suggesting these three families demonstrated ‘healthy family functioning’. In comparison to the study of adolescents with physical disabilities [24], the children in our study did not have any physical disabilities [1]; therefore, parents were not challenged by complex environmental barriers such as physical inaccessibility, specialized equipment, and access to transportation [45]. This allowed for immediate initiation of goals. Motivation and focus over this 4-week baseline may also have been facilitated by the biweekly check-ins from the therapist asking parents to rate their child's current performance for each goal. During this phase, parents demonstrated the ability to independently come up with solution-based environmental strategies that were effective, as found in other studies [46,47]. Further studies should include a larger, more socially diverse group of parents in terms of family functioning, while monitoring the specific strategies used by parents.

The initiative demonstrated by the parents, fueled by the therapist consultations and focus on environmental strategies, suggests the model of service delivery for this specific population may warrant further investigation. For example, early signs of restriction in participation were addressed very effectively with very few resources required. Addressing these issues at a young age may provide the foundation for development of more complex skills necessary to meet the greater environmental demands presented to all children in middle childhood and adolescence, and therefore be cost-effective in reducing their long-term need for services.

Children with a history of preterm birth are vulnerable to challenges in participation at an older age [4], therefore environmental interventions such as PREP hold promise in addressing these challenges at an early age in the child's natural environment and warrant some consideration [48]. Specifically, effective strategies for removing environmental barriers and enabling participation revealed in this study can inform practice and empower families.

### 4.1 Limitations

Common to single-subject research designs, this study involved a small sample size; however, each subject was studied intensely and involved a large number of repeated measures/observations, demonstrating clear-cut results. This design also allowed for replication of the intervention effect within different contexts (goals, families, environmental barriers), and, consequently, increases to a certain degree the generalizability of the findings [49]. Although our design exceeds the minimum criteria/requirements for replication (testing 3 participants x 3 goals each) [26], the original Interrupted Time Series (ITS) design with multiple baselines across participation goals [50] would have been more robust if it could have been practically implemented with this population.

Finally, the families in this study were categorized, based on the FAD, as ‘healthy/good’ in terms of family functioning and this may have affected the results. Further studies including more diverse families and including a qualitative component are required.

## 5 Conclusions

This study contributes to our understanding of therapeutic approaches applicable to children born preterm who do not exhibit a physical disability. Findings draw attention to the challenges of using specific methodological designs with motivated, self-directed families. The potential benefits of family-centered goal setting and the role parents play in enhancing their child's participation are highlighted. Results also suggest that a structured, goal-oriented environment-based approach, such as the PREP, may have the potential to improve the participation of these children in all settings. Larger studies in this underserved population are needed.

## Data availability

Access to data is restricted due to ethics concerns; in particular, child and family privacy.

## Funding

10.13039/501100000024Canadian Institutes of Health Research. 10.13039/501100001634Millennium Grant, National University of Ireland, Galway, Ireland.

## Declaration of competing interest

The authors declare that they have no known competing financial interests or personal relationships that could have appeared to influence the work reported in this paper.
